# *TNF-α* Polymorphisms in Major Depressive Disorder in Patients with and Without Cardiovascular Disease: A Systematic Review

**DOI:** 10.3390/biomedicines14040922

**Published:** 2026-04-17

**Authors:** Antonio Avelino Ferreira Soares, Yago Rodrigues Gontijo, Dante Mafra Tourino Teixeira, Bruna Rodrigues Gontijo, Alexandre Sampaio Rodrigues Pereira, Larissa Sousa Silva Bonasser, Caroline Ferreira Fratelli, Calliandra Maria de Souza Silva, Izabel Cristina Rodrigues da Silva

**Affiliations:** 1 Postgraduate Program in Health Sciences and Technologies, Faculty of Ceilândia, University of Brasília (UnB), Brasília-Federal District (DF), Brasília 72220-900, Brazil; aavelinofs@gmail.com (A.A.F.S.); yagorgontijoyrg@gmail.com (Y.R.G.); mafra.dante11@gmail.com (D.M.T.T.); brunargontijo.unb@gmail.com (B.R.G.); prof.alexandresampaio@gmail.com (A.S.R.P.); carolfratelli@gmail.com (C.F.F.); cdssilva@gmail.com (C.M.d.S.S.); 2 Clinical Analysis Laboratory, Molecular Pathology Sector, Pharmacy Department, Faculty of Ceilândia, University of Brasília (UnB), Brasília-Federal District (DF), Brasília 72220-900, Brazil; laribonasser@gmail.com; 3 Department of Pharmacy, Taguatina Campus, Centro Universitário Estácio de Sá, Taguatinga Sul Q CS CSG 9 Lotes 11/12/15/16, Taguatinga, Brasília-Federal District (DF), Brasília 72035-509, Brazil; 4 Postgraduate Program in Health Sciences, University of Brasília (UnB), Brasília-Federal District (DF), Brasília 70910-900, Brazil; 5 Academic Unit of Biotechnology Engineering (UAEB), Center for Sustainable Development of the Semi-Arid Region (CDSA), Sumé Campus, Federal University of Campina Grande, Sumé 58540-000, Brazil

**Keywords:** genetic polymorphism, major depressive disorder, tumor necrosis factor-alpha (TNF-α), pharmacogenetics, risk factors, inflammatory biomarkers, cardiovascular comorbidity, rs1800629 (−308 G/A), rs1799724 (−857 C/T), rs1799964 (−1031 T/C), antidepressant response, psychoneuroimmunology

## Abstract

**Introduction:** Major Depressive Disorder (MDD) has been increasingly associated with inflammatory dysregulation, particularly involving tumor necrosis factor-alpha (TNF-α). Genetic polymorphisms within the TNFA promoter region have been investigated as potential modulators of depressive susceptibility, symptom expression, treatment response, and inflammatory comorbidity. However, findings remain inconsistent across populations and clinical contexts. **Methods:** This systematic review adhered to PRISMA 2020 guidelines and was registered in PROSPERO (CRD420251242724). Observational and interventional studies evaluating associations between *TNFA* polymorphisms—specifically rs1800629 (−308 G/A), rs1799724 (−857 C/T), and rs1799964 (−1031 T/C)—and MDD-related outcomes in adults were included. Data extraction and methodological quality assessment were performed independently using an adapted GRIPS framework. **Results:** Eleven studies met the inclusion criteria, with eight investigating MDD without cardiovascular comorbidity and three assessing cardiovascular populations. Across diverse cohorts, rs1800629 and rs1799724 did not demonstrate consistent associations with MDD susceptibility. Although isolated population-specific findings were reported, genotype and allele distributions were generally comparable between cases and controls. Rs1799724 was associated with symptom dimensions and altered TNF-α expression in two cohorts. Rs1799964 was not linked to disease occurrence but showed potential association with antidepressant response and adverse cardiovascular outcomes in patients with chronic heart failure and comorbid depression. Overall, findings were heterogeneous and influenced by population characteristics, sample size, and clinical context. **Conclusions:** Current evidence does not support a robust etiological role for *TNFA* promoter polymorphisms in major depressive disorder. These variants may exert context-dependent modulatory effects on symptom expression, treatment response, or inflammatory-cardiovascular interactions rather than serving as primary susceptibility determinants. Larger, ethnically diverse studies integrating genetic, inflammatory, and clinical data are required to clarify the contribution of inflammatory genetic variability in depressive disorders.

## 1. Introduction

Major Depressive Disorder (MDD) is a prevalent psychiatric condition characterized by persistent low mood, anhedonia, cognitive dysfunction, and somatic symptoms, with significant functional impairment. Although psychosocial and environmental factors contribute to its development, increasing evidence supports the involvement of biological mechanisms, particularly immune-inflammatory pathways, in its pathophysiology [1,2,3].

MDD represents a major global health burden, affecting millions of individuals worldwide and significantly contributing to disability and suicide risk. The increasing prevalence observed in recent years reinforces the need to better understand its biological underpinnings and identify potential molecular markers associated with susceptibility and clinical variability [4].

One prominent hypothesis proposes that neuroinflammatory mechanisms contribute to MDD. Processes characterized by increased pro-inflammatory cytokines, such as interleukin-1β (IL-1β), interleukin-6 (IL-6) and tumor necrosis factor alpha (TNF-α), contribute to the dysregulation of the immune system by interfering with the neural circuits involved in mood regulation. Both central and peripheral inflammatory processes have been implicated in the onset and persistence of depressive symptoms [1,3].

These inflammatory signals may interfere with neuroendocrine regulation and favor the continuous activation of stress-related pathways, establishing a two-way interaction between the immune and neuroendocrine systems. Stressful stimuli activate the hypothalamus–pituitary–adrenal axis (HPA), promoting the release of corticotropin releasing hormone (CRH), adrenocorticotrophic hormone (ACTH) and glycocorticoids, which can be neurotoxic, besides influencing neuronal and glial cell function, particularly astrocytes and microglia, central mediators of the physiological response to stress and compromise neuroendocrine-immune homeostasis. Chronic activation of this system and persistent elevation of glycocorticoids can compromise neuronal function and affect brain regions involved in emotional regulation such as the hippocampus, amygdala and prefrontal cortex [1,3,5].

Among pro-inflammatory cytokines, tumor necrosis factor alpha (TNF-α) has been consistently investigated in MDD [1,3]. Elevated levels of peripheral TNF-α have been reported in depressed individuals, suggesting their potential role as a biomarker and pathophysiological mediator. There are still important gaps in understanding the biological mechanisms underlying individual vulnerability to inflammatory dysregulation in depression. In addition to circulating levels, genetic variability within the TNFA gene may influence cytokine expression and individual susceptibility to inflammatory phenotypes [5,6,7].

Although several studies have evaluated TNF-α promoter polymorphisms—particularly rs1800629 (−308 G/A), rs1799724 (−857 C/T), and rs1799964 (−1031 T/C)—results remain inconsistent [8,9,10]. Some investigations report associations with disease susceptibility, symptom severity, or treatment response, whereas others fail to detect significant genetic effects. Differences in sample size, ethnic background, clinical phenotype definition, and statistical modeling may contribute to these discrepancies.

Based on the inconsistencies observed in previous studies and the growing evidence linking inflammatory pathways to depressive disorders, this review aims to critically evaluate the association between inflammatory cytokine gene polymorphisms and depression. We hypothesize that specific genetic variants in inflammatory pathways are associated with depression in a context-dependent manner, influenced by clinical, demographic, and comorbidity-related factors.

## 2. Materials and Methods

### 2.1. Research Strategy and Selection Criteria

This systematic review was conducted in accordance with the Preferred Reporting Items for Systematic Reviews and Meta-Analyses (PRISMA 2020) statement (Table S1) and was prospectively registered in the International Prospective Register of Systematic Reviews (PROSPERO; registration number CRD420251242724).

Eligibility criteria were established a priori based on the PECOS framework (Population, Exposure, Comparison, Outcomes, and Study design). The Population comprised adult individuals (≥18 years) diagnosed with only Major Depressive Disorder (MDD) or studies that have at least separate data for MDD according to standardized diagnostic criteria (DSM or ICD) or validated structured clinical interviews. The Exposure of interest was genetic variation within the *TNFA* gene, with particular emphasis on promoter region polymorphisms, including rs1800629 (−308 G/A), rs1799724 (−857 C/T), and rs1799964 (−1031 T/C). However, other *TNFA* variants were considered eligible when reported in association with MDD-related outcomes.

The Comparison consisted of genotype and/or allele frequency distributions between individuals with MDD and non-depressed controls, or between clinically defined subgroups (e.g., treatment responders vs. non-responders; presence vs. absence of cardiovascular comorbidity). The predefined Outcomes included: (1) susceptibility to MDD; (2) symptom severity or dimensional phenotypes; (3) antidepressant treatment response; and (4) psychiatric outcomes in the context of cardiovascular disease. Eligible study designs included observational studies (case–control, cohort, or cross-sectional) and interventional studies conducted in human populations.

A comprehensive literature search was performed in the following electronic databases from inception to December 2025: MEDLINE (via PubMed), Web of Science, Scopus, Virtual Health Library (BVS), and the CAPES/MEC Portal. No restrictions regarding language or publication date were applied.

The search strategy combined Medical Subject Headings (MeSH) and free-text terms related to tumor necrosis factor-alpha, major depressive disorder, and genetic polymorphisms. Boolean operators were used to construct the search string as follows:

“TNF-α”, “Depressive Disorder, Major”, and “Polymorphism, Genetic”, combined using the Boolean operator “AND”. (TNFA OR “tumor necrosis factor alpha” OR TNF-alpha) AND (“Major Depressive Disorder” [MeSH] OR depression) AND (polymorphism OR SNP OR SNV OR variant) AND (rs361525 OR “−238” OR rs1799964 OR “−1031” OR rs1799724 OR “−863” OR rs1800630 OR “−863” OR rs3093662 OR “−851” OR rs3093665 OR rs1800629 OR “−308” OR rs1800610 OR “+489”).

Studies were excluded if they were reviews, meta-analyses, conference abstracts, animal or in vitro studies, or if they did not provide sufficient genotypic or allelic frequency data for MDD to allow interpretation of associations.

### 2.2. Selection of Studies and Data Extraction

All records identified through the database search were exported to reference management software for duplicate removal. The screening process was conducted in two sequential phases. In the first phase, two reviewers (AA and CS) independently screened titles and abstracts to assess eligibility according to the predefined PECOS criteria. Rayyan (Qatar Computing Research Institute) was used to facilitate blinded screening and conflict identification. In the second phase, the two reviewers independently evaluated full-text articles of potentially eligible studies to confirm inclusion. Any discrepancies between reviewers were resolved through discussion, and when consensus could not be reached, a third senior reviewer (IS) adjudicated the decision.

Data extraction was performed independently by two reviewers using a standardized, pre-piloted data collection form developed in Microsoft Excel. Extracted information included: first author, year of publication, country, study design, sample size, diagnostic criteria for MDD, population characteristics, genotyping method, evaluated *TNFA* polymorphisms, genotype and allele frequencies, statistical models applied, main outcomes, and reported *p*-values or effect estimates. For studies involving cardiovascular comorbidity, additional data were extracted regarding cardiac diagnosis, inflammatory biomarkers, clinical severity indices, and prognostic outcomes.

When relevant data were unclear or incomplete, information was interpreted directly from the published results. Automated data extraction tools were not employed.

### 2.3. Methodological Reporting Quality Assessment

The methodological quality and reporting adequacy of the included studies were assessed using the Genetic Risk Prediction Studies (GRIPS) statement. Although GRIPS was originally developed to improve transparency and completeness of reporting in genetic risk prediction studies, its structured framework allows for systematic appraisal of key methodological domains relevant to genetic association research.

The evaluation was conducted independently by two reviewers. Each of the 25 GRIPS items was assessed and categorized as adequately reported (score = 1) or insufficiently reported/not reported (score = 0). Total scores were calculated for each study to provide an overall estimate of reporting quality. Disagreements between reviewers were resolved through discussion, with arbitration by a third reviewer when necessary.

Studies achieving at least 75% of the total GRIPS items were considered to present adequate methodological reporting quality. This threshold was defined a priori to ensure consistency in evaluation.

Given the heterogeneity of the study designs and the absence of predictive modeling in several included studies, a quantitative formal bias risk tool specific for randomized or cohort studies was not applied. Differences in the genetic background of the population, sample size, diagnostic criteria, design of the study and the specific polymorphisms analyzed may contribute to the variability in reported results of genetic association studies in complex disorders such as major depressive disorder. In addition, some investigations have been conducted with very small sample sizes, which may reduce statistical power and increase the probability of type II error, thus limiting the detection of true genetic associations.

Possible sources of bias should be considered in interpreting the results of the included studies. Genetic association studies are particularly susceptible to bias related to population stratification, reduced or unbalanced sample sizes, and differences in diagnostic criteria or clinical instruments used to assess depression. In addition, variations in study design, genotyping methods and statistical approaches may influence the identification and interpretation of associations between TNF-α polymorphisms and major depressive disorder.

These methodological differences, together with the limited number of studies available for certain variants, may contribute to the inconsistency of the results reported in the literature. Thus, the findings should be interpreted with caution, since, considering the limitations inherent in observational studies of genetic association, inadequate correction can result in both underestimation and overestimation of genetic effects. depending on the analytical context and the relationship between ancestry, environment, population stratification, variability in sample size and multiplicity of statistical tests [11].

It is important to acknowledge that several included studies were based on relatively small sample sizes, which may increase the risk of random error and reduce statistical power. Small samples are more susceptible to both false-positive and false-negative findings, potentially contributing to inconsistencies across studies. Therefore, the interpretation of genetic associations should consider the variability introduced by sample size and population heterogeneity.

## 3. Results

### 3.1. Study Selection

The systematic search strategy yielded 81 records across the selected electronic databases. After removing 37 duplicate entries, 44 studies remained for title and abstract screening. Following independent evaluation of titles and abstracts against predefined eligibility criteria, 31 records were excluded. The main reasons for exclusion at this stage included the absence of genetic data related to *TNFA* polymorphisms, non-human study design, lack of specific assessment of major depressive disorder, and review-type publications.

Thirteen full-text articles were subsequently retrieved and assessed for eligibility. After detailed evaluation, two studies were excluded: one did not provide separate genotypic analysis for patients with MDD, as depressive and non-depressive psychiatric conditions were analyzed collectively, and one full-text article could not be accessed despite search attempts. Consequently, eleven studies fulfilled all inclusion criteria and were included in the final qualitative synthesis.

In this review, additional SNPs (rs361525 or −238, rs1800630 or −863, rs3093662 or −851, rs3093665 and rs1800610 or +489) of the TNF-α gene were included in the search strategy to broaden identification of potentially relevant variants. However, no studies have been found that specifically investigated these variants in human populations diagnosed with major depressive disorder (MDD) within the criteria established by the PECOS model. Thus, polymorphisms were excluded from the final analyses.

**Figure 1:**
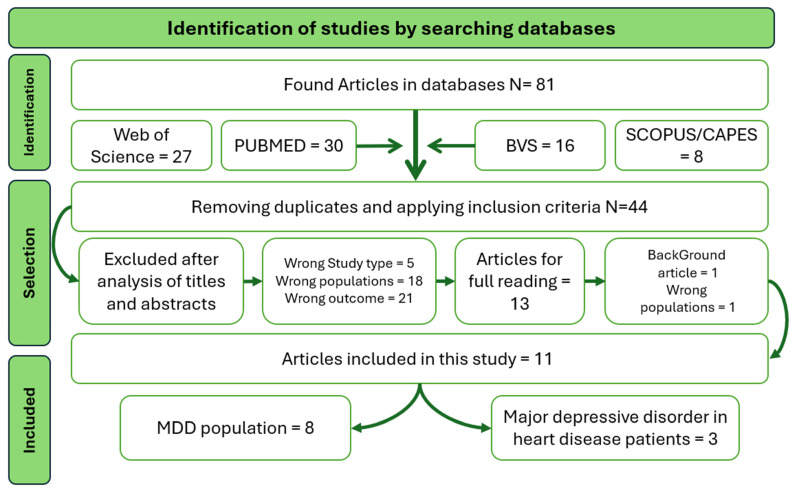
Flowchart of bibliographic research.

The detailed process of identification, screening, eligibility assessment, and inclusion is illustrated in Figure 1.

### 3.2. General Characteristics of Included Studies

The 11 included studies demonstrated considerable heterogeneity in geographic distribution, sample size, clinical characterization, and outcome definition. Eight studies investigated the association between *TNFA* polymorphisms and major depressive disorder in populations without cardiovascular comorbidity, whereas three studies evaluated patients presenting both cardiovascular disease and depressive symptoms. The included studies comprised both cross-sectional and longitudinal designs, which may have influenced the interpretation of temporal relationships between inflammatory markers and depressive outcomes. This distinction was considered during data synthesis to better contextualize the reported associations.

The eight studies focusing exclusively on MDD were conducted across multiple continents, including Asia (China, Iraq), Europe (Poland, Italy), North America (Canada), and Oceania (Australia). Sample sizes varied substantially, ranging from small cohorts of fewer than 40 participants to large case–control samples of more than 800 individuals diagnosed with MDD. Most studies employed case–control designs, although cross-sectional analyses and neuroimaging-based approaches were also represented. Diagnostic criteria were generally based on structured clinical interviews aligned with DSM classifications or validated symptom severity scales.

The principal characteristics of these eight studies, including genotype distributions, allele frequencies, statistical associations, and primary clinical outcomes, are summarized in Table 1.

The three studies addressing cardiovascular comorbidity evaluated populations with chronic heart failure, coronary heart disease, and acute coronary syndrome. These investigations extended beyond psychiatric diagnosis to include inflammatory biomarkers, cardiac functional indices, and prognostic outcomes. Their methodological characteristics and principal findings are detailed in Table 2.

A total of eight original studies evaluating the relationship between TNF-α gene polymorphisms and major depression, depressive symptoms, or related phenotypes were included, all published between 2009 and 2021. These studies were conducted across four continents, with Europe and Asia each contributing three studies (37.5%) [6,7,8,9,10,13], and North America and Oceania each contributing one study (12.5%) [12,14]. Among these, five studies (62.5%) employed a case–control design, two (25%) utilized a cross-sectional population design with dimensional symptom analysis, and one (12.5%) used a case–control design incorporating structural neuroimaging. 

Most of the included studies employed observational designs commonly used in genetic association research. Specifically, eight of the eleven studies (72.7%) used a case–control design [7,8,9,10,13,15,16], two studies (18.2%) adopted cross-sectional population designs assessing depressive symptoms or related phenotypes [12,14], and one study (9.1%) included a follow-up component in patients with cardiovascular disease, introducing a limited longitudinal perspective [17]. Overall, the evidence base is therefore predominantly derived from observational designs without prospective genetic risk modeling.

**Table 2:** Comparison between studies that evaluated the effect of TNF-α gene variants and Major Depressive Disorder in patients with cardiovascular diseases.

Lead Author	Country	Year	Title (Original/English)	Objective/Study Type	Sample Size (MDD)	Result Obtained	*p*-Value	Genotypic Frequency	Relationship Between Heart Problems and MDD Risk	Factors for Both Diseases
Opielak et al. [16]	Poland	2021	Effect of polymorphism rs1799964 in TNF-α gene on survival in depressive patients with chronic heart failure	Correlate rs1799964 (TNF-α) genotypes with clinical outcomes, cardiac/laboratory/nutritional parameters, and survival in patients with chronic heart failure (CHF) with depression. case–control study	66 chronic heart failure with depression (out of 94 CHF evaluated). 37 chronic heart failure without depression.	Genotype rs1799964 did not differ between depressed and non-depressed CHF patients. In depressed patients, CC was associated with lower EF%, worse NYHA, higher CRP/TNF-α, greater cachexia, and worse survival.	Ejection fraction(EF%) *p* = 0.023 NYHA examination *p* = 0.033 CRP *p* = 0.003 Cachexia incidence *p* = 0.017; TNF-α (pg/mL) *p* < 0.001.	TNFA-1031 rs1799964: CHF Depression (n = 66): C/C 10(15.1%) C/T 25(37.9%) T/T 31(47.0%) CHF Non-depression (n = 37): C/C 4(10.8%) C/T 13(35.1%) T/T 20(54.1%) HWE *p* = 0.202	Depression was frequent in CHF (70.2%) and is associated with a worse prognosis; the CC inflammatory genotype aggravated inflammation and the course of heart failure in depressed patients.	Inflammatory and nutritional axis: high CRP/TNF-α, anemia/low albumin and cachexia; cardiac factors: reduced EF; coexisting depression worsens quality of life and prognosis.
Kang et al. [17]	South Korea	2017	Genetic predisposition toward suicidal ideation in patients with acute coronary syndrome	Investigate genetic predisposition for suicidal ideation (SI) in patients with acute coronary syndrome (ACS) in the acute phase (≤2 weeks) and 1 year after ACS; to evaluate 10 polymorphisms (serotonergic, neurotrophic, carbon metabolism, and inflammatory cytokines). Cohort longitudinal study	ACS n = 969 (baseline)/711 (1 year); depression assessed by MINI (DSM-IV) in subgroup.	5-HTTLPR s allele associated with acute-phase SI after adjustment and Bonferroni; TNF-α −308G/A and IL-1β variants were associated in uncorrected analyses but lost significance after Bonferroni; no significant genetic association for SI at 1 year.	TNF-α −308G/A Baseline sample *p* = 0.168 TNF-α −308G/A Follow-up sample *p* = 0.983	TNFA-308 G/A rs1800629 (Baseline): No SI (N = 774): G/G 635(82.0%) G/A 125(16.1%), A/A 14(1.8%); SI (N = 195): G/G 146(74.9%) G/A 48(24.6%) A/A 1(0.5%) TNF-α −308G/A (Follow-up): No SI (N = 624): G/G 508(81.4%) G/A 107(17.1%) A/A 9(1.4%) SI (N = 87): G/G 69(79.3%) G/A 17(19.5%) A/A 1(1.1%) HWE *p* > 0,1	ACS is a serious cardiovascular condition associated with a higher risk of depression; SI was analyzed as a depressive symptom post-ACS.	For SI/post-ACS depression: female sex, lower education level, unemployment, living alone, personal/family history of depression, and baseline depression; for cardiovascular severity: Killip, biomarkers (troponin I/CK-MB), and cardiac events.
Golimbet et al. [15]	Russia	2017	Association of inflammatory genes with neuroticism, anxiety and depression in male patients with coronary heart disease (Cвязь гeнoв вocпaлитeльныx φaктopoв c нeвpoтизмoм, тpeвoжнocтью и дeпpeccиeй y мyжчин c ишeмичecкoй бoлeзнью cepдцa)	Examine the association of inflammatory polymorphisms (IL-4 –589C/T, IL-6 –174G/C, TNF-α –308G/A, CRP –717A/G) with depression and endophenotypes (neuroticism and trait anxiety) in men with coronary artery disease (CHD/IHD). case–control study	169 male patients with coronary heart disease. (91 individuals who did not have depression 78 patients with moderate to severe depression) 121 healthy controls	TNF-α –308G/A showed no association with coronary artery disease or with depression comorbid with coronary artery disease.	The *p*-values were not provided	TNFA-308 G/A rs1800629 MDD W/CHD: G/G 61(78.2%) G/A 15(19.2%) A/A 2 (2.6%) MDD no-CHD: G/G 70(76.9%) G/A 18(19.8%) A/A 3 (3.3%) Controls: G/G 96(75.6%) G/A 29(22.8%) A/A 2 (1.4%) Was in HWE no-mention *p*-value	Depression is common in CHD and worsens the course and quality of life; the authors propose inflammation as a shared mechanism between CHD and depression.	Systemic inflammation (IL-6, CRP, TNF-α) as a common pathway; genetic predisposition (IL-6/IL-4) may increase vulnerability to depression in patients with heart disease.

### 3.3. TNF-α −308 G/A (rs1800629)

The rs1800629 polymorphism was the most extensively investigated variant across the included studies, being evaluated in six of the eight MDD-only investigations. Despite its biological plausibility as a promoter-region variant that could influence TNF-α transcriptional activity, findings were heterogeneous across populations.

In the Polish study conducted by Jeleń et al. (n = 83 MDD cases; n = 248 controls), genotype frequencies among MDD patients were distributed as follows: G/G 69.9% (n = 58), G/A 30.1% (n = 25), and A/A 0%. In the control group, genotype frequencies were G/G 69.4% (n = 172), G/A 29.0% (n = 72), and A/A 1.6% (n = 4). Statistical analysis revealed no significant difference in genotype distribution (*p* = 0.5050) or allele frequencies (*p* = 0.7442), indicating virtual equivalence between cases and controls in this cohort.

Similarly, Białek et al. (n = 270 cases; n = 231 controls) reported genotype frequencies in responders and non-responders that did not differ significantly in disease occurrence (*p* = 0.616 for genotype; *p* = 0.644 for alleles). Among responders, the genotype distribution was A/A 1.3%, G/A 29.3%, and G/G 69.3%, whereas among non-responders, the distribution was A/A 1.4%, G/A 26.0%, and G/G 72.6%. These proportions demonstrate minimal absolute variation (<3%) between groups.

In contrast, Cerri et al. (n = 50 elderly MDD cases; n = 240 controls) observed statistically significant differences in genotype distribution (*p* = 0.007). In their case–control study, the G/G genotype was present in 84% of cases compared to 68.3% of controls, corresponding to an odds ratio of 2.433 (95% CI: 1.09–5.43). The A/A genotype was absent among cases but observed in 4.6% of controls. This finding suggests a potential age-specific association.

Kadhum et al. (n = 17 cases; n = 19 controls) reported genotype frequencies among cases of G/G 29.4%, A/G 64.7%, and A/A 11.8%, whereas controls demonstrated G/G 84.2% and A/G 15.8%, with no A/A genotype observed. Although genotype-level comparisons yielded *p*-values of 0.095 and 0.8009 for specific contrasts, allele frequency comparison revealed statistical significance (G vs. A, *p* = 0.0001). However, given the small sample size and wide proportional differences, these findings must be interpreted cautiously.

Large population-based studies, including McQuaid et al. (n = 469 genotyped participants) and Tartter et al. (n = 444), did not demonstrate significant interaction effects between rs1800629 and depressive symptom scores (*p*-values ranging from 0.08 to 0.87), further supporting lack of robust association in broader community samples in their cross-sectional studies.

Collectively, although isolated positive findings were observed, particularly in elderly or small samples, the majority of data do not support a consistent association between rs1800629 and MDD susceptibility.

### 3.4. TNF-α −857 C/T (rs1799724)

The rs1799724 polymorphism was examined in two independent Chinese Case–control studies with relatively large sample sizes.

Zhao et al. (n = 807 MDD cases; n = 822 controls) reported genotype frequencies among cases of T/T 5.9%, T/C 37.3%, and C/C 56.8%, compared to controls with T/T 5.6%, T/C 33.2%, and C/C 61.2%. Genotype distribution did not significantly differ between groups (*p* = 0.18), nor did allele frequency comparison (*p* = 0.11). However, when examining dimensional outcomes, T-allele carriers demonstrated significantly higher Hamilton Anxiety/Somatic subscale scores (*p* = 0.002) and higher TNF-α mRNA expression in the MDD group (*p* = 0.029), whereas overall TNF-α expression was elevated in MDD compared to controls (*p* < 0.01).

Zhou et al. (n = 144 MDD; n = 111 controls) similarly found no difference in genotype distribution (*p* = 0.766). However, neuroimaging analysis revealed a significant genotype × diagnosis interaction affecting gray matter volume in the right superior occipital gyrus, with voxel-level significance at *p* < 0.001 and cluster-level correction at *p* < 0.05 (AlphaSim corrected). T-allele carriers within the MDD group exhibited a greater reduction in cortical volume.

These findings consistently indicate absence of association with categorical diagnosis, but measurable effects on symptom dimensions and neuroanatomical correlates.

### 3.5. TNF-α −1031 T/C (rs1799964)

The rs1799964 polymorphism was evaluated in two Polish studies with differing conclusions regarding treatment response.

In Jeleń et al. (n = 83 cases; n = 248 controls), genotype frequencies among cases were T/T 66.3%, T/C 30.1%, and C/C 3.6%, compared to controls with T/T 67.3%, T/C 30.6%, and C/C 2.1%. Neither genotype (*p* = 0.7141) nor allele frequencies (*p* = 0.6961) differed significantly between groups.

Conversely, Białek et al. identified significant associations with antidepressant response. Among responders (n = 150), genotype distribution was T/T 62.7%, C/T 30.0%, and C/C 7.3%. Among non-responders (n = 73), T/T frequency increased to 82.2%, with C/T 16.4% and C/C 1.4%. The association between T/T genotype and poor response reached statistical significance (*p* = 0.004), and allele-level analysis similarly demonstrated significance for the T allele (*p* = 0.003). The absolute difference in T/T frequency between responders and non-responders exceeded 19 percentage points, suggesting a clinically relevant effect size within that cohort.

Despite these findings, replication remains limited, and disease susceptibility itself was not associated with this variant.

### 3.6. TNF-α Polymorphisms in the Context of Cardiovascular Disease

Three studies investigated the association between *TNFA* polymorphisms and depressive symptoms or related psychiatric outcomes in populations with established cardiovascular disease, encompassing patients with acute coronary syndrome (ACS), chronic heart failure (CHF), and coronary heart disease (CHD). These studies not only evaluated psychiatric endpoints but also incorporated clinical cardiological parameters and inflammatory biomarkers, thereby allowing a multidimensional analysis of genetic influence in complex comorbid contexts. The principal characteristics and statistical findings of these investigations are summarized in Table 2.

#### 3.6.1. rs1800629 (−308 G/A) in Cardiovascular Populations

The rs1800629 polymorphism was evaluated in two cardiovascular cohorts. In the large prospective study conducted by Kang et al., which included 969 patients assessed during the acute phase of ACS and 711 reassessed after one year, genotype distributions were analyzed in relation to suicidal ideation (SI), considered a clinically relevant depressive manifestation in this population.

At baseline, among patients without suicidal ideation (n = 774), genotype frequencies were as follows: G/G, 82.0% (n = 635); G/A, 16.1% (n = 125); and A/A, 1.8% (n = 14). Among those presenting suicidal ideation (n = 195), genotype distribution was G/G 74.9% (n = 146), G/A 24.6% (n = 48), and A/A 0.5% (n = 1). Although the proportion of heterozygous individuals (G/A) was numerically higher among patients with suicidal ideation (24.6% versus 16.1%), statistical analysis did not demonstrate a significant association after adjustment and Bonferroni correction (baseline *p* = 0.168).

At one-year follow-up, genotype distributions remained similar between groups. Among patients without suicidal ideation (n = 624), frequencies were G/G 81.4% (n = 508), G/A 17.1% (n = 107), and A/A 1.4% (n = 9), whereas in those with persistent or emergent suicidal ideation (n = 87), frequencies were G/G 79.3% (n = 69), G/A 19.5% (n = 17), and A/A 1.1% (n = 1). The association remained non-significant (*p* = 0.983). These findings indicate that although minor proportional differences were observed, particularly in heterozygous frequency, they did not translate into statistically robust associations when controlling for multiple testing.

Similarly, Golimbet et al. evaluated rs1800629 in a cohort of 169 male patients with coronary heart disease, of whom 78 presented moderate to severe depression, and 91 did not. Genotype frequencies among depressed CHD patients were G/G 78.2%, G/A 19.2%, and A/A 2.6%, while among non-depressed CHD patients, frequencies were G/G 76.9%, G/A 19.8%, and A/A 3.3%. In healthy controls (n = 121), genotype distribution was G/G 75.6%, G/A 22.8%, and A/A 1.4%. Although slight variations in A/A frequency were observed between groups (2.6% in depressed CHD versus 1.4% in controls), no statistically significant associations were reported. The authors noted that the limited sample size might have reduced statistical power to detect small genetic effects.

Collectively, these data do not demonstrate consistent evidence supporting rs1800629 as a determinant of depressive manifestations within cardiovascular disease populations.

#### 3.6.2. rs1799964 (−1031 T/C) in Chronic Heart Failure

In contrast to rs1800629, the rs1799964 polymorphism demonstrated clinically relevant associations in the context of chronic heart failure with comorbid depression in the study by Opielak et al.

Among patients with CHF and coexisting depression (n = 66), genotype distribution was C/C 15.1% (n = 10), C/T 37.9% (n = 25), and T/T 47.0% (n = 31). Among CHF patients without depression (n = 37), genotype distribution was C/C 10.8% (n = 4), C/T 35.1% (n = 13), and T/T 54.1% (n = 20). Although genotype frequencies did not differ significantly between depressed and non-depressed CHF groups in terms of psychiatric diagnosis per se, significant differences emerged when analyzing clinical severity and inflammatory parameters within the depressed subgroup.

Specifically, carriers of the C/C genotype exhibited a significantly lower left ventricular ejection fraction than carriers of other genotypes (*p* = 0.023), indicating reduced cardiac systolic performance. Furthermore, a worse New York Heart Association (NYHA) functional class was observed among C/C carriers (*p* = 0.033), suggesting more advanced symptomatic heart failure.

Inflammatory markers were also significantly elevated in C/C carriers. Circulating C-reactive protein levels were higher (*p* = 0.003), and plasma TNF-α concentrations demonstrated strong statistical significance (*p* < 0.001). Additionally, the incidence of cardiac cachexia was more frequent in C/C individuals (*p* = 0.017). Survival analysis indicated reduced survival probability among depressed CHF patients carrying the C/C genotype, although exact hazard ratios were not detailed in the summary table.

The magnitude and consistency of these associations across multiple clinical endpoints—cardiac function, inflammatory biomarkers, and survival—distinguish rs1799964 as the only variant among those studied to demonstrate coherent prognostic relevance within cardiovascular–psychiatric comorbidity.

#### 3.6.3. Synthesis of Cardiovascular Findings

When considering the three cardiovascular studies collectively, no consistent association was identified between *TNFA* promoter polymorphisms and the categorical presence of depressive diagnosis within cardiovascular disease populations. However, rs1799964 demonstrated statistically significant associations with markers of cardiac dysfunction and systemic inflammation in patients presenting both heart failure and depression.

These findings indicate that within cardiovascular contexts characterized by heightened inflammatory activity, specific *TNFA* variants may exert measurable influence on clinical severity and prognosis, rather than on primary susceptibility to depressive symptoms.

In this context, cardiovascular variables were considered as potential modifiers of the association between inflammatory gene polymorphisms and depression, rather than primary outcomes. Given the well-established link between cardiovascular disease, inflammation, and depressive symptoms, these factors may influence the observed genetic associations and should be interpreted as interacting components within a broader pathophysiological framework.

### 3.7. Quality Assessment

The methodological reporting quality of the included studies was evaluated using the adapted Genetic Risk Prediction Studies (GRIPS) framework, comprising 25 items covering key domains of study design, variable definition, genotyping procedures, statistical modeling, and transparency of reporting.

Across the eight studies evaluating MDD without cardiovascular comorbidity, GRIPS scores ranged from 17 to 23 out of 25 items, with a mean of 20.0 ± 3.0 and a median of 20.5 (Table S2). This distribution indicates moderate-to-high overall reporting completeness, with relatively limited dispersion between studies. Only one investigation scored below the predefined 75% adequacy threshold (i.e., fewer than 19 items fulfilled), although this study was retained because it met core eligibility criteria and provided extractable genotype data.

The three studies examining cardiovascular populations demonstrated similarly consistent reporting patterns, with scores ranging from 19 to 21 and a mean of 19.67 ± 1.15 (Table S2). The narrow standard deviation in this subgroup reflects a relatively homogeneous reporting structure among these investigations.

At the domain level, most studies adequately reported fundamental elements such as study design, participant eligibility criteria, diagnostic definitions, genotyping methodology, and primary statistical comparisons. Definitions of genetic variants followed standardized nomenclature in nearly all included articles, and genotyping techniques were generally described with sufficient detail to permit methodological interpretation.

However, variability was observed in specific methodological domains. In particular, the reporting of procedures for handling missing data was inconsistent across studies. While several investigations explicitly stated how incomplete datasets were managed, others did not clarify whether imputation, case exclusion, or sensitivity analyses were performed.

Similarly, formal validation of predictive or risk models—when applicable—was rarely reported. Only a minority of studies included any form of model validation or assessment of predictive performance metrics. Subgroup and interaction analyses were variably described, with some studies explicitly detailing exploratory analyses, whereas others did not specify whether additional stratified analyses were conducted beyond primary comparisons.

Another domain exhibiting heterogeneity concerned the reporting of risk distribution metrics and adjusted effect estimates. While most studies presented unadjusted genotype comparisons and corresponding *p*-values, fewer provided multivariable-adjusted effect sizes or confidence intervals. Measures of model fit and predictive ability were rarely detailed, particularly in studies focused solely on association rather than prediction.

Importantly, none of the included studies achieved full compliance with all 25 GRIPS items. The most commonly unfulfilled items related to explicit validation procedures, comprehensive reporting of predictive performance measures, and detailed description of model-building strategies.

Despite these limitations, the overall distribution of scores suggests that most studies provided sufficient methodological transparency to permit qualitative synthesis and interpretation of genetic associations. However, heterogeneity in reporting depth, statistical modeling approaches, and control of potential confounding variables underscores the methodological variability present in the available literature.

## 4. Discussion

The present systematic review synthesized and critically evaluated the available evidence regarding the association between TNFA gene polymorphisms and major depressive disorder (MDD), including clinical phenotypes, antidepressant response, and psychiatric manifestations in the context of cardiovascular comorbidity. Although inflammatory dysregulation is increasingly recognized as a relevant biological component of depression, the cumulative evidence derived from the included studies does not support a consistent or robust role for promoter-region TNFA polymorphisms as primary susceptibility determinants for MDD.

The inflammatory hypothesis of depression has been progressively refined in recent years. Contemporary models describe a cascade involving oxidative stress, reactive oxygen species (ROS), activation of microglia and astrocytes, and subsequent release of pro-inflammatory cytokines, including TNF-α, IL-6, and IL-1β [1]. This framework positions inflammatory signaling as a mediator capable of influencing synaptic plasticity, neurotransmission, and neural circuit integrity. Furthermore, systemic inflammation has been proposed as a biological interface linking MDD with comorbid medical conditions, particularly cardiovascular disease [3].

Peripheral biomarker studies provide additional empirical support for TNF-α involvement in depressive states. Elevated circulating TNF-α concentrations have been documented in adults with MDD compared to healthy controls [17], and inflammatory markers have been shown to correlate with symptom severity in validated scales such as the Montgomery–Åsberg Depression Rating Scale (MADRS) [15,16]. These data collectively reinforce the relevance of inflammatory activation in depressive phenotypes. However, elevated protein levels do not necessarily imply that common promoter polymorphisms within the *TNFA* gene exert a strong etiological influence on disease onset.

Among the variants examined, rs1800629 (−308 G/A) was the most extensively studied. Across multiple populations, larger case–control investigations frequently demonstrated nearly identical genotype and allele distributions between MDD patients and controls. Although isolated associations were observed in specific subgroups—most notably in elderly population—the lack of consistent replication across independent and ethnically diverse samples suggests that rs1800629 does not function as a universal genetic risk factor. Instead, any potential influence appears to be modest and context-dependent, potentially modulated by age, environmental exposures, or interaction with other biological pathways.

The rs1799724 (−857 C/T) polymorphism exhibited a similar pattern. While no consistent association was identified with categorical MDD diagnosis, it was linked to dimensional symptom expression and altered TNF-α mRNA levels in affected individuals. These findings are compatible with dimensional models of depression, in which inflammatory genetic variants may influence symptom severity or neurobiological endophenotypes without determining disease susceptibility per se [16].

The rs1799964 (−1031 T/C) variant did not demonstrate consistent association with MDD occurrence but showed potential relevance in two clinically distinct contexts. First, one study identified a significant association between the T/T genotype and reduced antidepressant response, suggesting a possible pharmacogenetic effect. Second, in patients with chronic heart failure and comorbid depression, the C/C genotype was associated with markers of worse cardiac function, elevated inflammatory biomarkers, increased cachexia, and reduced survival. These findings indicate that within biologically stressed systems characterized by heightened inflammatory activation, *TNFA* genetic variation may exert measurable effects on clinical severity and prognosis.

The cardiovascular subgroup analyses further support the relevance of inflammation as a shared biological pathway linking psychiatric and cardiac disorders [3]. In large cohorts of patients with acute coronary syndrome, rs1800629 did not demonstrate a significant association with suicidal ideation after correction for multiple comparisons. In contrast, rs1799964 exhibited consistent associations with inflammatory and prognostic markers in chronic heart failure, reinforcing the possibility that genetic effects become more evident in contexts of sustained systemic inflammation.

The discrepancy between robust evidence of increased TNF-α protein levels in depression [16] and the inconsistent associations observed for promoter polymorphisms underscores the complexity of inflammatory gene regulation. Cytokine expression is influenced by multilayered mechanisms, including epigenetic modifications and post-transcriptional regulation. Evidence indicating altered TNF-related regulatory pathways in suicide cases, including microRNA-mediated modulation, further illustrates that inflammatory gene activity may be governed by mechanisms not fully captured by single-nucleotide polymorphism analysis [17]. Thus, the absence of strong associations at the level of promoter polymorphisms does not invalidate the inflammatory hypothesis of depression but rather highlights the multifactorial and multilayered nature of its regulation [1,3].

Taken together, the findings of this review suggest that *TNFA* promoter polymorphisms are unlikely to function as strong, independent determinants of MDD risk. Rather, they may operate as modulatory factors whose influence becomes detectable under specific biological or clinical conditions, such as heightened inflammatory burden, pharmacological exposure, or cardiovascular comorbidity. This interpretation aligns with integrative models of depression that incorporate genetic susceptibility, immune activation, oxidative stress, and systemic health status into a unified framework [1,3].

The observed heterogeneity in genetic associations may be explained by complex biological mechanisms underlying depression. In addition to single-gene effects, depression is increasingly understood as a polygenic disorder influenced by the cumulative effect of multiple genetic variants. Furthermore, epigenetic modifications, such as DNA methylation and histone regulation, may modulate gene expression in response to environmental exposures. Gene–environment interactions, including stress, lifestyle factors, and comorbid conditions, likely play a critical role in shaping inflammatory responses and susceptibility to depression.

This review has several limitations that should be considered. First, the heterogeneity among included studies, including differences in study design, population characteristics, and diagnostic criteria, may have influenced the consistency of the findings. Second, the inclusion of studies with small sample sizes may have increased the risk of bias and reduced statistical robustness. Third, the predominantly observational nature of the included studies limits causal inference. Finally, variability in the assessment of depression and inflammatory markers across studies may have contributed to discrepancies in the reported associations.

Despite these limitations, the present review provides a structured synthesis of available genetic evidence and clarifies the current state of knowledge regarding *TNFA* polymorphisms in depression. By distinguishing between disease susceptibility, symptom modulation, pharmacogenetic effects, and cardiovascular prognostic relevance, this analysis contributes to a more nuanced understanding of the interface between inflammatory genetics and psychiatric outcomes. Future investigations integrating genomic, transcriptomic, and inflammatory biomarker data in adequately powered and ethnically diverse cohorts may further elucidate the clinical relevance of *TNFA* variation in depressive disorders and related comorbid conditions.

## 5. Conclusions

The present systematic review evaluated the association between TNFA gene polymorphisms and major depressive disorder, including clinical phenotypes, antidepressant response, and psychiatric manifestations in the context of cardiovascular comorbidity. The available evidence does not support a consistent or robust role for promoter-region TNFA variants—particularly rs1800629 (−308 G/A), rs1799724 (−857 C/T), and rs1799964 (−1031 T/C)—as primary susceptibility factors for MDD.

Across diverse populations and study designs, genotype and allele distributions were generally comparable between individuals with MDD and controls. Although isolated associations were identified in specific subgroups, these findings were not consistently replicated across independent cohorts. Notably, certain variants demonstrated associations with intermediate phenotypes, including symptom dimensions, inflammatory markers, treatment response, and cardiovascular prognostic markers, suggesting a context-dependent, modulatory rather than etiological role.

In cardiovascular populations characterized by heightened inflammatory burden, rs1799964 showed associations with markers of cardiac dysfunction and adverse clinical outcomes, suggesting that the clinical impact of TNFA variation may be more pronounced in biologically stressed systems. However, replication remains limited, and the strength of evidence does not support clinical implementation at this stage.

Overall, TNFA promoter polymorphisms appear unlikely to function as strong independent determinants of major depressive disorder. Future research should prioritize adequately powered, ethnically diverse cohorts with standardized phenotypic characterization and integrative approaches combining genetic, inflammatory, and clinical data to clarify the potential contribution of inflammatory genetic variability within depressive disorders and related comorbid conditions.

In conclusion, the findings suggest that inflammatory gene polymorphisms may be associated with depression, although these associations appear to be context-dependent and influenced by multiple biological and environmental factors. Future studies with larger, well-characterized samples and standardized methodologies are needed to clarify these relationships and improve the understanding of the genetic contribution to depression.

## Figures and Tables

**Table 1:** Comparison between studies that evaluated the effect of TNF-α gene variants polymorphisms and Major Depressive Disorder.

Lead Author	Country	Year	Title	Objective/Study Types	Sample Size MDD (n)	Result Obtained	*p*-Value	Genotypic Frequency	
Aamal Muhsen Kadhum [8]	Iraq	2021	Associations of Tumor Necrosis Factor–α G-308A (rs1800629) Gene Polymorphisms with Major Depressive Disorder	Analyze TNF-α gene polymorphisms in major depressive disorder using allele-specific PCR. Case–control study	MDD Case N° 17 Control N° 19	There was a weak association between TNF-α genotypes and MDD, but a significant association with allele frequency. Genotypes had a weak impact on MDD, but the distribution of alleles may have a potential role in the disease.	Genotypic frequency: GG: *p* = 0.095, GA: *p* = 0.8009 and AA: *p* = 0.8009 Allelic frequency: HWE (G vs. A) *p* = 0.0001	TNFA-308 G/A rs1800629 Cases MDD G/G = 29.4% (n = 5) G/A = 64.7% (n = 11) A/A = 11.8% (n = 2) Control G/G = 84.2% (n = 16) A/G = 15.8% (n = 3)	
Margaret Tartter [12]	Australia	2015	Effects of Chronic Interpersonal Stress Exposure on Depressive Symptoms are Moderated by Genetic Variation at IL6 and IL1β in Youth	Investigate functional SNPs in IL6 (−174G>C), IL1β (−511C>T) and TNF (−308G>A) as moderators of the relationship between chronic stress and increased depressive symptoms in young people.Cross-sectional study	N° 444 Australian young adults W/depressive symptoms	No major effects of TNF were found, nor was there a relevant moderation effect of TNF on the reported depressive outcome.	TNF x chronic interpersonal stress *p* = 0.11 Depressive symptoms in response to non-interpersonal stress *p* = 0.08 TNF × non-interpersonal stress: *p* = 0.57 TNF × interpersonal stress: *p* = 0.10		
Agnieszka Jeleń [13]	Poland	2021	Preliminary investigation of two promoter region polymorphisms of the TNFA gene in patients with recurrent depressive disorder	Determine the frequency of TNFA G−308A (rs1800629) and T-1031C (rs1799964) SNPs in patients with recurrent depression and to assess their association with the development/progression and response to treatment.Case–control study	MDD Case 83 (M:25, F:58)Control 248	No significant difference in genotypes/alleles between cases and controls for G-308A and T-1031C; there was also no association with clinical/therapeutic variables.	G-308A genotypes *p* = 0.5050; alleles *p* = 0.7442. T-1031C genotypes *p* = 0.7141; alleles *p* = 0.6961. Therapeutic response (HDRS change): G−308A *p* = 0.109; T-1031C *p* = 0.1361. HWE *p* > 0.05	TNFA−308 G/A rs1800629 G/G 58 (69.9%) G/A 25 (30.1%) A/A 0 Controls G/G 172 (69.4%) G/A 72 (29.0%) A/A 4 (1.6%)	TNFA−1031 T/C rs1799964: T/T 55 (66.3%) T/C 25 (30.1%) C/C 3 (3.6%) Controls T/T 167 (67.3%) T/C 76 (30.6%) C/C 5 (2.1%)
Katarzyna Bialek [10]	Poland	2020	Preliminary Study of the Impact of Single-Nucleotide Polymorphisms of IL-1α, IL-1β and TNF-α Genes on the Occurrence, Severity and Treatment Effectiveness of the Major Depressive Disorder	Test 5 SNPs (IL1α/IL1β/TNF-α: rs1143623, rs1143627, rs17561, rs1799964 -1211T>C—TNF-α, rs1800629 488G>A—TNF-α) with risk, severity (HDRS) and response to antidepressants; to evaluate gene-gene interactions.Case–control study	MDD Case N° 270 Control N° 231	Identifies polymorphisms associated with severity and treatment response; rs1799964 is associated with a low response (TT and T allele).	Therapeutic response Treatment TNFA-1031 T/C rs1799964: *p* = 0.001 TT *p* = 0.004; CT *p* = 0.032; T allele *p* = 0.003. 488G>A no association with response *p* = 0.616 and alleles *p* = 0.644.HWE *p* > 0.05	TNFA-1031 T/C rs1799964: 150 Responsive T/T 94 (62.7%) C/T 45 (30.0%) C/C11 (7.3%) 73 Non-responsive TT 60 (82.2%) CT 12 (16.4%) CC 1 (1.4%)	TNFA−308 G/A rs1800629 150 Responsive A/A 2 (1.3%) G/A 44 (29.3%) G/G 104 (69.3%) 73 Non-responsive A/A 1 (1.4%) G/A 19 (26.0%) G/G 53 (72.6%)
Junxiong Zhao [7]	China	2020	Identification of TNFA influencing MDD risk and clinical features in Han Chinese	Compare peripheral expression of TNF-α mRNA between MDD and controls; to test the association of rs1799724 with MDD risk; to evaluate the association of rs1799724 with clinical characteristics and TNF-α expression.Case–control study	MDD Case N° 807 Control N° 822	MDD showed higher TNF-AMF mRNA than controls; rs1799724 was not associated with MDD risk (genotypic/allelic NS distribution), but the T-allele (TT/TC) was associated with higher somatic score and higher TNF-AMF mRNA in patients.	Genotype case–control *p* = 0.18; alleles *p* = 0.11; TNFA mRNA (MDD vs. control) *p* < 0.01; TNFA mRNA by genotype in MDD *p* = 0.029; HAMD anxiety/somatic *p* = 0.002; total HAMD *p* = 0.008.HWE *p* > 0.05Case *p* = 0.88Control *p* = 0.27	TNFA-857 T/C rs1799724 Cases: T/T 48 (5.9%) T/C 301 (37.3%) C/C 458 (56.8%) Controls: T/T 46 (5.6%) T/C 273 (33.2%) C/C 503 (61.2%)	
Rubai Zhou [6]	China	2018	Effects of tumor necrosis factor-α polymorphism on the brain structural changes of the patients with major depressive disorder	Test whether the rs1799724 SNP (TNFA) contributes to neuroanatomical changes (gray matter volume) in patients with MDD and its diagnosis/genotype interaction.Case–control study	MDD Case N° 144 M:60 F:84 Control N° 111 M:53 F:58	There was no difference in the rs1799724 genotypic distribution between MDD and controls; however, there was a diagnosis × genotype interaction affecting the volume of the visual cortex (right superior occipital gyrus), with a greater reduction in MDD carriers of the risk genotype (T-carriers).	Genotype between groups *p* = 0.766; Total brain volume (ml) *p* = 0.240 and VBM analyses reported with voxel *p* < 0.001 and cluster *p* < 0.05 (AlphaSim).HWE *p* = 0.806	TNFA-857 T/C rs1799724 Cases: C/C 110 (76.4%) C/T+T/T 34 (23.6%); Controls: C/C 83 (74.8%) C/T+T/T 28 (25.2%).	
Anna Paola Cerri [9]	Italy	2009	The −308 (G/A) single nucleotide polymorphism in the TNF-α gene and the risk of major depression in the elderly	Investigate the association of TNF-α −308(G/A) with late-onset major depression in elderly individuals without dementia.Case–control study	MDD Case N° 50 Control N° 240	Genotypic and allelic distribution differed between groups; E was more frequent in cases and associated with a higher risk (OR = 2.433).	Genotype *p* = 0.007; Alleles *p* = 0.05; OR GG = 2.433 (95%CI 1.09–5.43).Was in HWE no-mention *p*-value	TNFA-308 G/A rs1800629 Cases: G/G 42 (84%) G/A 8 (14.6%) A/A 0; Controls: G/G 164 (68.3%) G/A 65 (27.1%) A/A 11 (4.6%)	
Robyn Jane McQuaid [14]	Canada	2019	Understanding the Relation Between Early-Life Adversity and Depression Symptoms: The Moderating Role of Sex and an Interleukin-1β Gene Variant	Examine whether the SNPs IL-1β rs16944, IL-6 rs1800795, and TNF-α rs1800629 (−308 G/A) moderate the relationship between early adversity and depressive symptoms; to assess moderation by sex.Cross-sectional study	N° 475 university studentsCross-sectional study	The TNF-α rs1800629 did not have significant interactions in the moderation model.	TNF-α: Childhood maltreatment *p* = 0.87 Depression scores *p* = 0.76	TNFA-308 G/A rs1800629 (N = 469 genotyped): G/G 324 (69.1%) G/A 133 (28.4%) A/A 12 (2.6%)	

## Data Availability

The data used in the review are those from the article’s bibliographic references. Articles that met the inclusion criteria were analyzed according to the Genetic Risk Prediction Studies (GRIPS) guideline to determine their quality. We used other bibliographic references only to discuss or clarify points from the selected articles presented in Table 1 and Table 2.

## Supplementary Materials

The following supporting information can be downloaded at: https://www.mdpi.com/article/10.3390/biomedicines14040922/s1, Table S1: PRISMA_2020_checklist_TNF-alfa and TDM; Table S2: Quality Evaluation of articles according to the adapted GRIPS guideline [18].
