# Supplementary material for: TNF-α Polymorphisms in Major Depressive Disorder in Patients with and Without Cardiovascular Disease: A Systematic Review
Source: Biomedicines. 2026 Apr 17;14(4):922. doi: 10.3390/biomedicines14040922 (PMC13113129; doi:10.3390/biomedicines14040922)
Supplement: Supplementary file 1 [file biomedicines-14-00922-s001.zip › Table S2.pdf]

biomedicines

MDPI

Table S2. Quality Evaluation of articles according to the adapted GRIPS guideline.

Section/Topic		Kang et al., 2017 [17]	Golimbet et al., 2017 [15]	Opielak et al., 2021 [16]	Cerri et al., 2009 [9]	Kadhum et al., 2021 [8]	Zhou et al., 2018 [6]	Zhao et al., 2020 [7]	Tartter et al., 2015 [12]	Jeleń et al., 2021 [13]	Białek et al., (2020) [10]	McQuaid et al., 2019 [14]
**Title and Resume**												
**Title**	1)Identifies study as genetic risk.	x	x	x	X	x	x	x	x	x	x	x
**Introduction**												
**Clarity**	2) Clear scientific rationale	x	x	x	X	x	x	x	x	x	x	x
**Objective**	3) Clearly defined objectives	X	X	X	X	X	X	X	X	X	X	X
**Methods**												

**Study design and setting**	4) Present key elements of study design early in the paper and describe the setting, locations, and relevant dates, including periods of recruitment, exposure, follow-up, and data collection.	x	x	x	X	x	x	x	x	x	x	x
**Participants**	5) Describe eligibility criteria for participants, and sources and methods of selection of participants.	x	x	x	x	x	x	x	x	x	x	x
**Variables: Definition**	6) Clearly define all participant characteristics, risk factors and outcomes. Clearly define genetic variants using a widely-	x	x	x	x	x	x	x	x	x	x	x

	used nomenclature system.											
**Variables: Assessment**	7) (a) Describe sources of data and details of methods of assessment (measurement) for each variable.	x	x	x	x	x	x	x	x	x	x	x
	(b) Give a detailed description of genotyping and other laboratory methods.	x	x	x	x	x	x	x	x	x	x	x
**Variables: Coding**	8) (a) Describe how genetic variants were handled in the analyses	x	x	x		x	x	x	x	x	x	x
	(b) Explain how other quantitative variables were handled in the analyses. If applicable,	x	x	x		x	x	x	x	x	x	x

	describe which groupings were chosen, and why.											
**Analysis: Risk model construction**	9) Specify the procedure and data used for the derivation of the risk model. Specify which candidate variables were initially examined or considered for inclusion in models. Include details of any variable selection procedures and other model building issues. Specify the horizon of risk prediction (e.g., 5-year risk).	x			x	x	x	x	x	x		x
**Analysis: Validation**	10) Specify the procedure and data used for								x			x

	the validation of the risk model.											
**Analysis: Missing data**	11) Specify how missing data were handled.	X	X	X	x	x	x	x	x	x	X	x
**Analysis: Statistical methods**	12) Specify all measures used for the evaluation of the risk model including, but not limited to, measures of model fit and predictive ability.	x	X	x	x	x	x	x	x	x	X	x
**Analysis: Other**	13) Describe all subgroups, interactions, and exploratory analyses that were examined.	X	X	x			x	x	x		x	x
**Results**												
**Participants**	14) Report the numbers of individuals at each stage of the study. Give	x		x	x	x	x	x	x	x		x

	reasons for non-participation at each stage. Report the number of participants not genotyped, and reasons why they were not genotyped.										x	
**Descriptives: Population**	15) Report demographic and clinical characteristics of the study population, including risk factors used in the risk modeling.	x	x	x	x	x	x	x	x	x	x	x
**Descriptives: Model estimates**	16) Report unadjusted associations between the variables in the risk model(s) and the outcome. Report adjusted estimates and their precision	x	X	x	x	x	x	x	x	x		x

	from the full risk model(s) for each variable.											
**Risk distributions**	17) Reports the predicted risk distribution and/or its scores.	x		x		x	x	x	x	x		x
**Assessment**	18) Report measures of model fit and predictive ability, and any other performance measures, if pertinent.	x		x					x			x
**Validation**	19) Report any validation of the risk model(s).											
**Other analyses**	20) Present results of any subgroup, interaction, or exploratory analyses, whenever pertinent.	x	x	x	X		x	x	x		x	x
	**Discussion**											

**Limitations**	21) Discuss limitations and assumptions of the study, particularly those concerning study design, selection of participants, and measurements and analyses, and discuss their impact on the results of the study.	x	x	x	x	x	x	x	x	x	X	x
**Interpretation**	22) Give an overall interpretation of results considering objectives, limitations, multiplicity of analyses, results from similar studies, and other relevant evidence.	x	x	x	x	x	x	x	x	x	x	x
**Generalizability**	23) Discuss the generalizability			x	x	x	x	x	x	x	X	x

	and, if pertinent, the health care relevance of the study results.											
**Supplementary data**	24)There is additional data on other risk factors or conditions.	X					X		X			X
**Financing / conflicts Feedback**	25)There is information regarding funding and conflicts of interest.	X	X	X	X	X	X	X	X	X	X	

Present: X.
